# Enhancing the Biological Properties of White Chocolate: *Moringa oleifera* Leaf Extract as a Natural Functional Ingredient

**DOI:** 10.3390/foods14030359

**Published:** 2025-01-22

**Authors:** Sandra M. Gomes, Rita Miranda, Lúcia Santos

**Affiliations:** 1 LEPABE—Laboratory for Process Engineering, Environment, Biotechnology and Energy, Faculty of Engineering, University of Porto, Rua Dr. Roberto Frias, 4200-465 Porto, Portugal; scgomes@fe.up.pt; 2 ALiCE—Associate Laboratory in Chemical Engineering, Faculty of Engineering, University of Porto, Rua Dr. Roberto Frias, 4200-465 Porto, Portugal; 3 FEUP—Faculty of Engineering, University of Porto, Rua Dr. Roberto Frias, 4200-465 Porto, Portugal; up201907337@edu.fe.up.pt

**Keywords:** *Moringa oleifera*, antioxidants, functional foods, white chocolate

## Abstract

*Moringa oleifera* tree is recognised for its high content of bioactive compounds. This work explored the potential of incorporating its leaves or respective extracts into white chocolate to enhance its biological and sensory properties as white chocolate lacks the beneficial compounds found in cocoa. In this study, a phenolic-rich extract was obtained from *Moringa oleifera* leaf powder, and its biological properties and phenolic composition were characterised. The extract displayed good antioxidant capacity, especially against ABTS radical (IC_50_ = 162.0 mg/L). Additionally, it exhibited strong inhibitory potential against α-amylase and β-glucosidase, achieving average inhibition rates of 79.9% and 98.0%, respectively. The main phenolic compounds identified included catechin (0.211 mg_compound_/g_extract_), caffeic acid (0.056 mg_compound_/g_extract_), and quercetin (0.031 mg_compound_/g_extract_). White chocolate samples were fortified with 1% and 3% *M. oleifera* leaf extract, resulting in increased antioxidant properties and oxidative stability. All formulations were microbiologically safe, and the sample containing 3% extract showed the highest DPPH inhibition after 15 days of storage and a higher delay in the autoxidation of lipids over time. The fortification of white chocolate with *M. oleifera* leaf extract has the potential to transform it into a functional product rich in antioxidants, providing health benefits and increased value.

## 1. Introduction

Chocolate is one of the most consumed delicacies in the world. Its main categories, dark, milk, and white, differ in the content of three ingredients: cocoa solid, milk fat, and cocoa butter [1]. To produce them, cacao beans from the *Theobroma cacao* tree are the primary raw material. This crop is grown under specific climate and biophysical conditions, with African countries and tropical regions of Asia and Latin America being the primary cacao-producing countries. To generate chocolate, cacao beans go through an extraction process, along with several other procedures, where a paste containing cocoa solid and cocoa butter is obtained. Cocoa powder results from the elimination of cocoa butter from the mixture, and it is the main ingredient for the production of dark chocolate. On the other hand, white chocolate (WCh) is made of cocoa butter, thus lacking the bioactive and phenolic compounds associated with cacao [2]. Therefore, WCh is regarded as an unhealthier version of chocolate, and, with proper fortification, it could be turned into a functional product with increased nutritional value and beneficial health effects [3].

In the food industry, the maintenance of sensory characteristics and the shelf life of products are two relevant concerns, usually solved by food additives. Their use is strictly regulated by governmental organisations, being regularly subject to alterations due to its controversial nature. *Moringa oleifera* (MO) is one of many plants that can be used both as a functional food and as a natural food additive [4].

**Figure 1:**
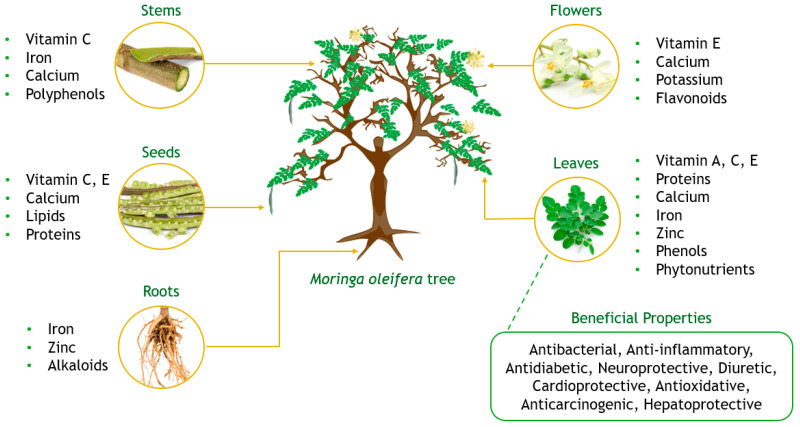
Composition of different parts of the *Moringa oleifera* tree.

*M. oleifera* is a tropical tree belonging to the Moringaceae family, which includes 13 different species. Its history dates to 150 B.C., when it was claimed that monarchs consumed their leaves and fruits to maintain mental alertness and healthy skin. It has also been reported that ancient Mauritanian warriors drank *M. oleifera* leaf extract (MOLE) as an elixir for extra energy and to relieve pain during war [5]. This tree is native to the sub-Himalayan region in the north of India, Pakistan, Africa, Asia Minor, and Arabia and has been introduced to several parts of the world [6]. MO is widely referred to as the “miracle tree” or “tree of life”, recognising its beneficial effects on human nutrition and health, as well as its distinguished bioactive properties [7]. The consumption of this plant has been described to contribute significantly to fighting malnutrition due to the high distribution of phytochemicals and bioactive compounds (BACs) in each part of the plant and their high nutritional value [8]. Most parts of the MO tree are edible and can provide multiple benefits for the consumer [5]. Figure 1 shows an overview of the main compounds present in each part of the *M. oleifera* tree and the range of the medicinal effects of their leaves.

*M. oleifera* leaves are an excellent source of beneficial compounds for the human diet, such as proteins, vitamins, and phytonutrients such as polyphenols, carotenoids, tocopherols, and ascorbic acid [9], making them an exceptional ingredient to fortify food products.

To date, research conducted on the incorporation of MOLE into sweet food products is scarce. Furthermore, this extract is also not linked to studies with chocolate, which brings value and novelty to this work. To the best of the authors’ knowledge, fortified food studies associated with WCh are scarce and recent, which, allied to its low functionality, makes it a good candidate for enrichment with bioactive compounds. As a result, the inclusion of MOLE, particularly from their leaves, as the food fortificant inWCh, could be a sustainable option to improve its nutritional quality, bioactive properties, and overall shelf life. Moreover, the possibility of using MOLE to manage diabetes was evaluated by assessing the extract’s capacity to inhibit enzymes linked to carbohydrate digestion. This is particularly important after consuming carbohydrate-rich foods, such as white chocolate. Therefore, the objective of the present work was to evaluate the possibility of using MOLE as a natural ingredient in white chocolate to improve its biological activity and extend the product’s shelf life.

## 2. Materials and Methods

### 2.1. Samples and Reagents

The *Moringa oleifera* leaves were collected in Luanda, Angola (8°57′24.9″ S, 13°13′02.9″ E). The *Moringa oleifera* leaf powder (MOLP) was provided by Agostinho Neto University (UAN), after drying and grounding the collected leaves. The obtained powder presented a particle size below 250 μm. The cocoa butter, powdered milk, and powdered sugar, used in the production of white chocolate, were purchased from a supermarket in Porto, Portugal.

Ultrapure water was obtained using a Milli-Q water purification equipment from Merck Millipore (Burlington, MA, USA), and ethanol (Ref. 83813.360, C_2_H_6_O, CAS 64-17-5) was bought from VWR (Radnor, PA, USA).

For analysis of the total phenolic content, Folin–Ciocalteu reagent (Ref. 47641) from Sigma-Aldrich (St. Louis, MO, USA) and sodium carbonate (Ref. 13418, CNa_2_O_3_, CAS 497-19-8) from Honeywell (Charlotte, NC, USA) were used. For the antioxidant capacity, 2,2-diphenyl-1-picrylhydrazyl (Ref. D9132, C_18_H_12_N_5_O_6_, CAS 1898-66-4), 2,2′-azino-bis(3-ethylbenzothiazoline-6-sulphonic acid) (Ref. A1888, C_18_H_24_N_6_O_6_S_4_, CAS 30931-67-0), (±)-6-hydroxy-2,5,7,8-tetramethylchromane-2-carboxylic acid (Ref. 238813, C_14_H_18_O_4_, CAS 53188-07-1), and potassium persulphate (Ref. 379824, K_2_S_2_O_8_, CAS 7727-21-1) were acquired from Sigma-Aldrich, and acetic acid (Ref. 20104.312, C_2_H_4_O_2_, CAS 64-19-7) and sodium acetate (Ref. 27653.260, C_2_H_3_NaO_2_, CAS 127-09-3) were bought from VWR (Radnor, PA, USA). For the anti-enzymatic analyses, α-amylase (Ref. A3176), starch (Ref. S4180, C_6_H_10_O_5_, CAS 9005-25-8), and β-glucosidase kit (Ref. MAK129) from Sigma-Aldrich and dihydrogen phosphate dihydrate (Ref. 28015.261, NaH_2_PO_4_·2H_2_O, CAS 13472-35-0) and di-sodium hydrogen phosphate anhydrous (Ref. 28026.260, Na_2_HPO_4_, CAS 7558-79-4) from VWR were used. For the quantification of the phenolic compounds, acetonitrile (Ref. 83639.320, C_2_H_3_N, CAS 75-05-8) and twelve analytical standards were purchased from Sigma-Aldrich: chlorogenic acid (Ref. C3878, C_16_H_18_O_9_, CAS 327-97-9), gallic acid (Ref. 91215, C_7_H_6_O_5_, CAS 149-91-7), catechin (Ref. C1251, C_15_H_14_O_6_, CAS 154-23-4), kaempferol (Ref. 60010, C_15_H_10_O_6_, CAS 520-18-3), caffeic acid (Ref. C0625, C_9_H_8_O_4_, CAS 331-39-5), epicatechin (Ref. E1753, C_15_H_14_O_6_, CAS 490-46-0), ellagic acid (Ref. E2250, C_14_H_6_O_8_, CAS 476-66-4), ferulic acid (Ref. PHR1791, C_10_H_10_O_4_, CAS 537-98-4), quercetin (Ref. Q4951, C_15_H_10_O_7_, CAS 117-39-5), rosmarinic acid (Ref. 536954, C_18_H_16_O_8_, CAS 20283-92-5), resveratrol (Ref. R5010, C_14_H_12_O_3_, CAS 501-36-0), and procyanidin B2 (Ref. 42157, C_30_H_26_O_12_, CAS 29106-49-8).

For the production of chocolate, lecithin (Ref. L0023, CAS 8002-43-5) was obtained from TCI (Tokyo, Japan). For the microbial analyses, agar (Ref. J637, CAS 9002-18-0) from VWR and Rose Bengal Chloramphenicol Agar (Ref. 1.00467.0500) and m-Lauryl Sulphate Broth (Ref. 0734) from Merck (Darmstadt, Germany) were used. For lipid oxidation tests, ammonium thiocyanate (Ref.A10632, CH_4_N_2_S, CAS 1762-95-4) was bought from Alfa Aesar (Haverhill, MA, USA), barium chloride dihydrate (Ref. 217565, BaCl_2_·2H_2_O, CAS 10326-27-9) and *p*-anisidine (Ref. A88255, C_7_H_9_ON, CAS 104-94-9) were obtained from Sigma-Aldrich, and isooctane (Ref. 28780.322, C_8_H_18_, CAS 540-84-1), chloroform (Ref. 22711.244, CH_3_Cl, CAS 67-66-3), methanol (Ref. 20864.320, CH_3_OH, CAS 67-56-1), and iron sulphate (II) heptahydrate (Ref. 24244.232, FeSO_4_·7H_2_O, CAS 7782-63-0) were bought from VWR.

### 2.2. Extraction of Phenolic Compounds from M. oleifera Leaf Powder

The phenolic compounds present in MOLP were extracted by solid–liquid extraction, using a Soxhlet extractor, for 2 h. Ethanol was used as extraction solvent, with a sample–solvent ratio of 1:20 (m/V). The solvent was removed from the samples using a rotary evaporator (BÜCHI Rotavapor R-200, Flawil, Switzerland), with a bath temperature of 35 °C and a pressure of 90 mbar. Total evaporation of the solvent was achieved by subjecting the samples to a constant stream of nitrogen at a pressure of 2 mbar. The extracts were protected from light and stored at 4 °C until further use.

### 2.3. Characterisation of the M. oleifera Leaf Extract

#### 2.3.1. Total Phenolic Content

The total phenolic content (TPC) of the extract was determined using the Folin–Ciocalteu method, according to the literature [7]. The measurements were performed in quadruplicate.

#### 2.3.2. Antioxidant Capacity

The antioxidant capacity of the extract was determined by evaluating its ability to inhibit two free radicals: 2,2-diphenyl-1-picrylhydrazyl (DPPH) and 2,2′-azinobis(3-ethyl-benzothiazolin-6-sulphonic acid) (ABTS). The assays were performed according to the literature, with some modifications [7]: for DPPH, extract solutions ranging from 1500 mg/L to 15,000 mg/L were prepared; for ABTS, the concentration of the extract solutions ranged from 250 mg/L to 2500 mg/L. The inhibition percentage of the radicals was calculated, and a calibration curve of the percentage of radical inhibition vs. the extract concentration was obtained to determine the necessary concentration of extract to inhibit 50% of the radical (IC_50_) using Equation (1) where b represents the intersection of the calibration curve on the y-axis and m represents the slope. The antioxidant power of the extract was also compared to a standard antioxidant, Trolox. The standard concentration ranged from 0.05 mg/L to 10 mg/L for DPPH and 0.05 mg/L to 5 mg/L for ABTS. The results were expressed as mg of Trolox equivalents (TE)/g of extract. All measurements were performed in quadruplicate.

(1) $${\mathrm{IC}}_{50}\;\left(\mathrm{mg}/L\right)=\frac{50\;-\;b}{m}$$

#### 2.3.3. Analysis of the Inhibitory Potential Towards α-Amylase and β-Glucosidase

The inhibitory potential of MOLE towards two enzymes was studied. For α-amylase, the protocol employed by Ferreira et al. [10] was used, with slight modifications. Briefly, 250 μL of a 1 mg/mL sample solution (in ethanol) was mixed with 250 μL of a 0.1 mg/mL α-amylase solution (in ultrapure water) and incubated for 10 min at 37 °C. Then, 250 μL of 1% *w*/*v* starch solution (in phosphate buffer, 0.02 M, pH 6.9) was added and incubated for 10 min at 37 °C. Later, 500 μL of 3,5-dinitrosalicylic acid (DNS) reagent was added, followed by a 5 min incubation in boiling water. After cooling down to room temperature, 5 mL of water was added to the solution. The absorbance was read at 540 nm, in triplicate, and the percentage of inhibition was calculated according to Equation (2), where Abs_sample_ refers to the absorbance of the extract and Abs_control_ refers to the absorbance of the control, which was obtained by substituting the extract solution for ethanol.

(2) $$\alpha -\mathrm{amylase}\;\mathrm{inhibition}\;\left(\%\right)=\frac{{\mathrm{Abs}}_{\mathrm{control}}\;-\;{\mathrm{Abs}}_{\mathrm{sample}}}{{\mathrm{Abs}}_{\mathrm{control}}}\;\times \;100$$

Regarding the β-glucosidase assay, it was performed according to its respective technical bulletin [11]. In a 96-well plate, 20 μL of sample solution (1 mg/mL in a 0.02 mM phosphate buffer, pH 6.9) was mixed with 200 μL of Master Reaction Mix (200 μL buffer:8 μL β-NPG substrate). The initial absorbance of the samples was measured at 405 nm (Abs_initial_). The plate was incubated at 37 °C for 20 min, and the final absorbance of the samples was measured (Abs_final_). A control and a blank were also prepared by mixing 20 μL of distilled water with 200 μL of the calibrator or distilled water, and their absorbance was measured at 405 nm after the 20 min incubation (Abs_calibrator_ and Abs_water_, respectively). The β-glucosidase activity was calculated according to Equation (3), and the β-glucosidase inhibition percentage was calculated according to Equation (4), where βGA_control_ is the β-glucosidase activity of the control (250 units/L), and βGA_sample_ is the activity of the samples calculated from Equation (3).

(3) $$\beta -\mathrm{glucosidase}\;\mathrm{activity}\;\left(\mathrm{units}/L\right)=\frac{{\mathrm{Abs}}_{\mathrm{final}}\;-\;{\mathrm{Abs}}_{\mathrm{initial}}}{{\mathrm{Abs}}_{\mathrm{calibrator}}\;-\;{\mathrm{Abs}}_{\mathrm{water}\;}}\;\times \;250\;\mathrm{units}/L$$

(4) $$\beta -\mathrm{glucosidase}\;\mathrm{inhibition}\;\left(\%\right)=\frac{{\beta \mathrm{GA}}_{\mathrm{control}}\;-\;{\beta \mathrm{GA}}_{\mathrm{sample}}}{{\beta \mathrm{GA}}_{\mathrm{control}}}\times \;100$$

#### 2.3.4. Identification and Quantification of Phenolic Compounds by HPLC-DAD

The phenolic compounds in MOLE were quantified by HPLC-DAD using the external standard method, as described by Gomes et al. [7]. The presence of twelve different phenolic compounds was evaluated. Their identification was based on the compounds’ retention time, and the quantification was performed at the maximum absorption wavelength, specific for each compound: 222 nm for catechin, epicatechin, and procyanidin B2; 255 nm for ellagic acid; 275 nm for gallic acid; 305 nm for resveratrol; 322 nm for caffeic acid, chlorogenic acid, and ferulic acid; 330 nm for rosmarinic acid; and 365 nm for kaempferol and quercetin. The results were expressed as mg of phenolic compound/g of extract. The measurements were performed in triplicate.

### 2.4. Incorporation of M. oleifera Leaf Extract in White Chocolate

#### 2.4.1. White Chocolate Production and Formulation

To produce white chocolate (WCh), 12 g of cacao butter was melted using a double boiler on medium to low heat. Once melted, 6 g of powdered sugar was added and stirred until total dissolution. Powdered milk was added, depending on the formulation (as described in Table 1), and the mixture was poured into a food processor (Qilive Coffee Grinder Q5321, Croix, France) for 1 min. Then, the chocolate was transferred into the mould and placed in the freezer, at −4 °C for 2 h. Finally, the chocolate was stored in the fridge (4 °C) in a closed container and protected from the light for one week and then moved to room temperature for three weeks.

Four different WCh formulations were produced to evaluate the performance of *Moringa oleifera* leaf extract (MOLE) as a natural preservative: a chocolate without additives was produced as negative control (WCh-NC); a positive control containing soy lecithin, a synthetic commonly used emulsifier and preservative in white chocolate (WCh-PC); and two formulations containing MOLE at different substitution levels, 1% *w*/*w* and 3% *w*/*w* (WCh-MOLE 1% and WCh-MOLE 3%, respectively). Soy lecithin was added at a concentration of 0.1%, the typical amount used in commercial chocolate. Each formulation was produced in duplicate.

The samples were stored over 1 month, and analyses were performed on the week of production (t_0_), 5 days after storing at room temperature (t_1_), and 15 days after storing at room temperature (t_2_).

#### 2.4.2. Microbiological Analysis of the White Chocolates

To assure their microbiological safety after production and throughout the study, 0.5 g of each WCh was placed in saline solution (0.9% NaCl) and vortexed for 1 min. Subsequently, 100 μL of each solution was spread, in duplicate, on Lauryl Sulphate Agar (LSA) and Rose Bengal Chloramphenicol Agar (RBC), specific mediums for the growth of coliform microorganisms, and yeast and moulds, respectively. The LSA plates were left to incubate for 24 h at 37 °C and the RBC plates for 7 days at 25 °C. After incubation, the colony-forming units (CFUs) were enumerated.

#### 2.4.3. Antioxidant Capacity of the White Chocolates

To extract the phenolic compounds from WCh, 4 mL of ethanol was added to 2 g of each formulation. These solutions were homogenised in a vortex for 1 min and placed in an ultrasound bath (VWR USC, Radnor, PA, USA) for 5 min. This procedure was performed three times. Then, the solutions were centrifuged for 10 min at 3000 rpm, and the supernatant was collected. The extraction process was repeated two times, and the obtained solutions were used to determine the DPPH radical scavenging activity of the WChs as described in Section 2.3.2.

#### 2.4.4. Oxidative Stability of the White Chocolates

To evaluate the primary and secondary lipid oxidation of the white chocolate over time, the peroxide value (PV) and *p*-anisidine value (*p*-AV) were determined, respectively, following the protocol presented by Ferreira and Santos [12], with slight alterations.

For the PV, an iron chloride solution was firstly prepared: 50 mL of a barium chloride solution (8 mg/mL in water) was gently added to 50 mL of an iron sulphate solution (10 mg/mL in water) under agitation; afterwards, 2 mL of HCl 10 M was added to the filtered solution. For the assay, 0.5 g of melted chocolate was dissolved in 9.8 mL of chloroform–methanol (7:3 *v*/*v*). Then, 50 μL of an ammonium thiocyanate solution (300 mg/mL in water) was added, followed by the addition of 50 μL of the iron chloride solution and 4 s of vortex. The samples were incubated for 5 min at room temperature, protected from light, and the absorbance was measured at 500 nm. Equation (5) was used to calculate the PV, where Abs_sample_ refers to the sample absorbance, Abs_blank_ refers to the absorbance of the blank prepared without WCh sample, and m_sample_ refers to the mass of the WCh sample used.

(5) $$\mathrm{PV}=\frac{\left({\mathrm{Abs}}_{\mathrm{sample}}\;-\;{\mathrm{Abs}}_{\mathrm{blank}}\right)\;\times \;12.25}{m_{\mathrm{sample}}\;\times \;55.84\;\times \;2}$$

For determining the *p*-AV, the protocol was followed as described by Ferreira and Santos [12]. Firstly, 1 g of each WCh sample was diluted in 25 mL of isooctane, and the absorbance was measured at 350 nm. Afterwards, 5 mL of each solution was transferred to another falcon tube, 1 mL of *p*-anisidine reagent (2.5 mg/mL in acetic acid) was added, and the solutions were left to incubate for 10 min, after which the absorbance was measured at 350 nm. The *p*-AV value was determined using Equation (6), where Abs_AR_ is the absorbance of the sample after the reaction with the *p*-anisidine reagent, Abs_BR_ is the absorbance of the sample before the reaction with the *p*-anisidine reagent, and m_sample_ is the mass of the WCh sample added.

(6) $$p-\mathrm{AV}=\frac{25\;\times \;(1.2\;\times \;{\mathrm{Abs}}_{\mathrm{AR}}\;-\;{\mathrm{Abs}}_{\mathrm{BR}})}{m_{\mathrm{sample}}}$$

Finally, the total oxidation value (TOTOX) was calculated to obtain a global representation of the oxidation of the chocolates by applying Equation (7).

(7) TOTOX=2PV + p-AV

### 2.5. Statistical Analysis

To verify the statistical significance of the obtained data, a two-factor analysis of variance (2-way ANOVA) was performed, using GraphPad Prism 8.0.2. A *p*-value inferior to a significance level of 0.05 was considered a statistically significant value within a confidence interval of 95%. Results were presented as mean ± standard deviation.

## 3. Results and Discussion

### 3.1. Characterisation of M. oleifera Leaf Extract

In the present study, phenolic-rich extracts were obtained, aiming their incorporation into white chocolate to produce a value-added product. The Soxhlet technique was selected to obtain the phenolic compounds from *Moringa oleifera* leaf powder (MOLP). This method utilises the principle of reflux and siphoning to continuously extract the product with fresh solvent, which has the advantage of requiring less solvent and being simple to operate. However, the high temperatures needed may increase the possibility of the thermal degradation of the extracted solutes [13]. Ethanol was selected as the extraction solvent due to its relatively low boiling point, which allows for energy consumption reduction, and its compatible polarity with phenolic compounds (PCs). This solvent also has a GRAS (Generally Recognised as Safe) status and is approved by the Food and Drug Administration (FDA), making it suitable for food applications. To evaluate the potential benefits of incorporating *Moringa oleifera* leaf extract (MOLE) in white chocolate, the chemical and biological properties of the obtained extract were assessed.

The Folin–Ciocalteu reagent was used to determine the total phenolic content (TPC) of the extract in an alkaline environment, which allows the protons present in phenolic compounds to dissociate into phenolic ions. The greater the phenolic concentration, the more phenolic ions will reduce the heteropolyacid, resulting in a more concentrated blue colour and a higher value of absorbance of the sample [14]. The TPC obtained for the MOLE was (38.6 ± 5.0) mg_GAE_/g_DW_. This value is inferior to the ones in the literature for multiple extraction conditions. For instance, Gomes et al. [7] obtained a TPC of (54.5 ± 16.8) mg_GAE_/g_DW_ for ultrasound-assisted solid–liquid extraction, with 70% ethanol as solvent. Additionally, Tai et al. [15], using mechanical agitation for 24 h with 80% ethanol to extract the phenolic compounds from MOLP, obtained a TPC of (74.9 ± 0.2) mg_GAE_/g_DW._ This result may indicate that other methods could be more effective for the extraction of phenolic compounds. Nevertheless, it is important to confirm if higher TPCs are translated to superior biological properties of interest.

The antioxidant capacity of MOLE (against DPPH and ABTS radicals) and its potential to inhibit the action of different enzymes (α-amylase and β-glucosidase) were analysed. The results obtained are summarised in Table 2.

From Table 2, it is possible to conclude that both radicals were inhibited by MOLE. A higher antioxidant capacity was verified towards ABTS as the extract’s concentration required to inhibit 50% of this radical (IC_50_) is inferior, and the corresponding TEAC (the comparison of the antioxidant potential with a standard antioxidant, Trolox) is higher. This result is corroborated by multiple studies on MOLE. Regarding the literature with equivalent extraction conditions, Ferreira et al. [10] obtained IC_50_ values of (544.0 ± 7.9) mg/L for DPPH and (115.2 ± 4.9) mg/L for ABTS, while Gomes et al. [16] presented results of (636.0 ± 9.2) mg/L for DPPH and (205.2 ± 4.6) mg/L for ABTS. In this assay, the results were higher for DPPH, which could be influenced by a different geographical origin of the MO leaves. In fact, factors like climatic conditions, time of year, soil type, and altitude may lead to variations in the chemical composition of the leaves, which can impact their antioxidant activity as well as the type and quantity of PCs [17]. These disparities can be reflected in the results of the TPC, DPPH, and ABTS assays.

Different enzymes are responsible for the catabolism of starch, glycogen, and disaccharides in the gastrointestinal tract, such as α-amylase and β-glucosidase. Their inhibition is usually linked to the management of diabetes by decreasing the rate of carbohydrate metabolism, which controls blood glucose levels [18]. These enzymes have a complementary function, acting on distinct substrates and breaking down different glycosidic bonds. While α-amylase is involved in the initial stages of carbohydrate digestion, degrading large polysaccharides into oligosaccharides or disaccharides, β-glucosidase has a role in the last steps, converting disaccharides and glycosides into glucose [19]. It is possible to observe, from Table 2, that the MOLE was capable of inhibiting both enzymes, particularly β-glucosidase. Other studies also suggest that MOLE can effectively inhibit the enzymes responsible for carbohydrate metabolism. Ferreira et al. [10] reported an α-amylase inhibition of (94.1 ± 0.4)%, while Magaji et al. [18] obtained an inhibitory activity of MO aqueous leaf extract of (40.7 ± 0.4)% for α-glucosidase. Therefore, this assay shows the relevance of incorporating MOLE in a sweet product, such as white chocolate, since it could inhibit the degradation of carbohydrates after ingestion.

The presence of phenolic compounds is tightly related to the beneficial properties of MO. An HPLC-DAD analysis was performed to identify and quantify the main phenolic compounds contained in the MOLE. The results are described in Table 3. Catechin was the major phenolic compound identified, while chlorogenic acid, ellagic acid, epicatechin, ferulic acid, kaempferol, resveratrol, and rosmarinic acid were not detected in the obtained extract.

It is relevant to notice that the PCs identified, as well as their concentrations, can vary amongst the literature due to differences in the origin of *Moringa oleifera* leaves or in the extraction conditions applied. Nevertheless, Gomes et al. [7] also suggested the prevalence of catechin in MOLE, with a concentration of 19.83 mg_compound_/g_extract_, and indicates a residual presence of caffeic acid and quercetin. These results are in accordance with the ones obtained in the present work. However, other PCs, such as epicatechin or chlorogenic acid, have been detected by this author, even though they were not identified in this research. A possible explanation could be the use of a different extraction technique, which could influence the compounds present in the extract. Regarding gallic acid, various authors, like Hassan et al. [20], have reported this PC as one of the major compounds present in MOLE, with concentrations reaching 105.67 mg/g. In the analysis performed, even though this compound was identified in the chromatogram, it was not quantifiable. A possible explanation for this discrepancy could reside in the fact that gallic acid is a very polar molecule and, therefore, difficult to extract with alcohol or hydroalcoholic solutions with low water content. Although a mixture of ethanol and water could enhance the extraction efficiency of phenolic compounds, the mixture of the two solvents can present some disadvantages: first, while ethanol can be evaporated using only the rotary evaporator, the addition of water requires an extra step of lyophilisation, which poses additional energetic and economic costs; second, mixing water and ethanol generates azeotropes, which makes it impossible to reuse the extraction solvent. Nevertheless, the obtained extract exhibited promising antioxidant capacity, and the ability to inhibit the activity of α-amylase and β-glucosidase enzymes, making it an interesting candidate for the fortification of white chocolate.

### 3.2. Characterisation of the Fortified White Chocolates

**Figure 2:**
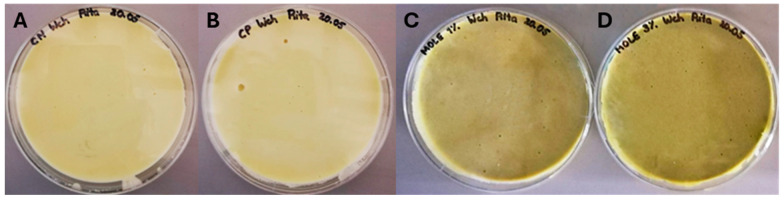
White chocolate samples the day after production. (**A**) WCh-NC (white chocolate negative control); (**B**) WCh-PC (white chocolate positive control); (**C**) WCh-MOLE1% (white chocolate with *M. oleifera* leaf extract at a substitution level of 1%); and (**D**) WCh-MOLE3% (white chocolate with *M. oleifera* leaf extract at a substitution level of 3%).

To understand the effect of the incorporation of MOLE into white chocolate (WCh), four WCh samples were produced: a negative control without any fortification (WCh-NC), a positive control with 0.1% lecithin, a typical emulsifier and preservative used in the chocolate industry (WCh-PC), and fortified samples with the incorporation of 1% and 3% of MOLE (WCh-MOLE1% and WCh-MOLE3%, respectively). The produced white chocolates can be seen in Figure 2. Visually, in terms of colour, the WCh-NC and WCh-PC samples presented a similar tone of beige, while with the incorporation of MOLE the chocolates exhibited a greenish tone. As expected, a higher incorporation percentage of MOLE in the WCh originated a more intense green tonality, which was confirmed by Poliński et al. [21] regarding the incorporation of MOLP. In terms of texture, the WCh-PC presented a harder consistency than the WCh-NC, possibly due to the better emulsification of the ingredients caused by the addition of lecithin. The MOLE-incorporated WChs also presented a slightly harder consistency than WCh-NC.

It is relevant to mention that, during the production process, the WCh was not able to be tempered. Tempering is a crystallisation technique, performed at up to 32 °C, that helps to stabilise the cocoa butter crystals during storage [21]. It can influence the appearance of the chocolate in terms of shininess and colour and also its resistance to heat variations and overall shelf life [3].

#### 3.2.1. Microbiological Analysis of the White Chocolates

To ensure that the consumption of the WChs produced does not compromise human safety, the existence of microbial contaminations in the formulations was evaluated on the week of production. The assay was performed using two selective mediums: Lauryl Sulphate Agar (LSA) was used to evaluate coliform microorganisms, while Rose Bengal Chloramphenicol Agar (RBC) assessed contamination from moulds and yeasts. The results of the assay are presented in Figure S1 of the Supplementary Material. After the incubation period, no colonies were observed in any sample, except for WCh-NC in the RBC medium. However, the number of colonies detected was inferior to 30, which is considered negligible. Hence, the incorporation of the MO phenolic extracts did not interfere with the microbial safety of the product.

#### 3.2.2. Antioxidant Capacity of the White Chocolates

**Figure 3:**
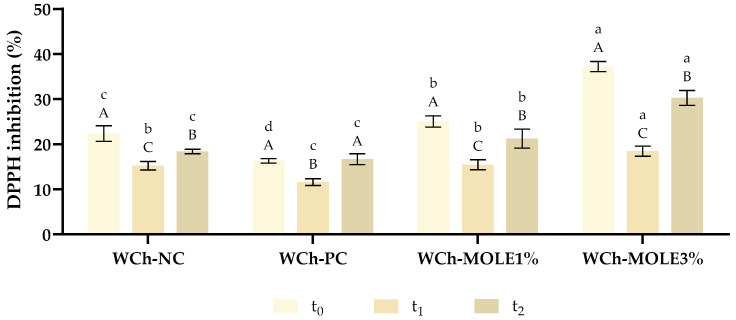
Variation of the antioxidant capacity towards DPPH of the white chocolate samples throughout the period of study. The analyses were performed at different timepoints: the week of production, stored in the fridge (t_0_), 5 days after storing at room temperature (t_1_) and 15 days after storing at room temperature (t_2_). WCh-NC—white chocolate negative control; WCh-PC—white chocolate positive control; WCh-MOLE1%—white chocolate with *M. oleifera* leaf extract at a substitution level of 1%; and WCh-MOLE3%—white chocolate with *M. oleifera* leaf extract at a substitution level of 3%. For the same timepoint, different small letters (a–d) represent statistically different values for different white chocolate samples (*p* < 0.05). For the same white chocolate sample, different capital letters (A–C) represent statistically different values for different timepoints (*p* < 0.05).

To assess the potential of MO extract in extending the shelf life of white chocolate, while enriching this product in phenolics and antioxidants, the DPPH assay was performed. The evolution of the DPPH radical inhibition was studied over 15 days, and the obtained results are presented in Figure 3.

Comparing the WCh samples, in the week of production (t_0_), the incorporation of MOLE resulted in a higher DPPH inhibition than the other samples, with WCh-MOLE3% demonstrating the highest value since it has a higher concentration of antioxidant compounds in its formulation. As expected, WCh-NC and WCh-PC presented the lowest DPPH inhibition since the first was not fortified with bioactive compounds and the second contains soy lecithin, which is mostly composed of phospholipids and other fatty acids, and does not contain PCs in its composition [22].

For t_1_, a significant reduction in the DPPH inhibition was verified in all samples. Although WCh-MOLE3% remained the sample with the highest inhibition of DPPH radical, it was also the one that exhibited the most pronounced decrease. This decrease could be attributed to the degradation of polyphenols caused by exposure to external factors such as light, humidity, heat, or oxygen [10]. Therefore, it is necessary to protect the phenolic compounds incorporated, using, for example, microencapsulation, to maintain the bioactive potential of MOLE throughout time.

Contrarily to the established trend, from t_1_ to t_2_, an increase in the inhibition of this radical was observed. Throughout their storage, some compounds present in the WCh might have suffered degradation and oxidation, which could interfere with the results of these assays since during lipid oxidation free radicals are produced [23]. Additionally, it is relevant to mention that the analysed period of 15 days is not considered sufficient for a proper analysis since the expiration date of WCh is usually higher, reaching several months or years if correctly stored [24].

#### 3.2.3. Oxidative Stability of the White Chocolates

The evolution of the shelf life of white chocolate is mostly affected by lipid oxidation and non-enzymatic browning reactions, such as the Maillard reaction [25]. In this product, the Maillard reaction is characterised by chemical interactions between the milk proteins and the aldehyde groups of the reducing sugars (lactose), resulting in the deterioration of the milk powder used in its formulation [24]. This reaction generates multiple reaction products and water, and, since white chocolate is a product with low water activity, these browning reactions tend to occur slower due to the limited mobility of the reactants. However, they are favoured by higher concentrations of proteins and reducing sugars, which is verified in WCh [25]. Hence, its high composition in fats and the absence of antioxidants can result in both lipid oxidation and non-enzymatic browning reactions, which are involved in the production of off flavours, the browning of milk solids, and the evolution of its colour, as well as the decrease in nutritional quality and shelf life of the product. As a result, it is essential to control these reactions by the addition of antioxidants to suppress the formation of free radicals, avoiding the oxidation of lipids and food spoilage [24].

**Figure 4:**
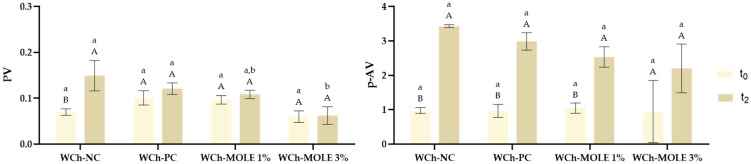
Variation of the peroxide value (left) and the *p*-anisidine value (right) of the white chocolate samples over time. The analyses were performed the week of production, stored in the fridge (t_0_), and 15 days after storing at room temperature (t_2_). WCh-NC—white chocolate negative control; WCh-PC—white chocolate positive control; WCh-MOLE1%—white chocolate with *M. oleifera* leaf extract at a substitution level of 1%; and WCh-MOLE3%—white chocolate with *M. oleifera* leaf extract at a substitution level of 3%. For the same timepoint, different small letters (a, b) represent statistically different values for different white chocolate samples (*p* < 0.05). For the same white chocolate sample, different capital letters (A, B) represent statistically different values for different timepoints (*p* < 0.05).

To evaluate the oxidative stability of the WCh samples, their peroxide value (PV) and *p*-anisidine value (*p*-AV) were determined at the beginning and end of the experimental period. The PV is associated with the production of hydroperoxides, the main byproducts of the oxidation of oil and fats, which are related to the oxidation propensity of free fatty acids [12]. When hydroperoxides break down, secondary oxidation products such as carboxyl acids, aldehydes, and ketones are produced. The presence of these compounds is measured by the *p*-AV assay. A compound named *p*-anisidine reacts intensely with aldehydes, producing molecules that absorb light at 350 nm, the equivalent to the absorbance of a solution where an oil or fat reacts with *p*-anisidine in an iso-octane environment. To analyse the overall oxidative stability of the white chocolate formulations, the total oxidation value (TOTOX) was determined. This parameter is important since the rate of hydroperoxide generation is not necessarily associated with the production rate of secondary oxidation products, i.e., a high PV does not equate to a high *p*-AV. The lipid oxidation results are displayed in Figure 4 and Table S1 of the Supplementary Material.

At the first timepoint (t_0_), all the WCh samples presented statistically similar PV and *p*-AV values as the oxidation of the lipids is not expected to occur immediately after production. The TOTOX values (Table S1) ranged from 1.07 to 1.24 mEq O_2_/kg fat. After storing the white chocolates for 15 days (t_2_), the PV values remained stable. However, a significant increase (*p* < 0.05) was observed in the *p*-AV values for all samples except WCh-MOLE3%. As anticipated, WCh-NC showed the highest increase. This increase affected the TOTOX values, with WCh-MOLE3% presenting the lowest value (2.33 mEq O_2_/kg fat) and WCh-NC presenting the highest value (3.73 mEq O_2_/kg fat). Although the large variation observed for the WCh-MOLE3% in the *p*-AV analysis may affect the discussion of the results, the authors believe that the globality of the results give some preliminary information on the effect of the addition of the MOLE extract in the lipid oxidation of the white chocolate, indicating that the addition of MOLE successfully slowed down the autoxidation of lipids in WCh, as reported by Feihrmann et al. [26], who evaluated the incorporation of MOLE in beef, and by Salem et al. [27], who concluded that the use of MOLE as preservative in sour cream decreased its PV. Also, an increase in extract concentration led to a slower increase in the oxidation value. A similar finding was reported by Ferreira and Santos [12], who studied the incorporation of a phenolic-rich extract from avocado peels into mayonnaise. An increase in the oxidation values within just 15 days may be attributed to the high temperatures present during the season when the experiments were conducted. Elevated temperatures can accelerate the natural oxidation of lipids, leading to the formation of peroxides [24].

Rossini et al. [24] and Vercet [25] investigated the evolution of browning and oxidation in WCh during storage by measuring its PV. In both studies, the initial PV for WCh with or without additives was approximately 2.0 mEq O_2_/kg fat. In contrast, the samples analysed in this research showed PV values ranging from 0.06 to 0.10 mEq O_2_/kg fat. This disparity may be attributed to variations in the methodologies used to evaluate PV, as well as the quality of the ingredients and chocolates produced. Rossini et al. [24] verified an increase in this parameter, during 10 months of storage, to around 6.9 mEq O_2_/kg fat for the control, and Vercet [25] reached a maximum PV of 9.5 mEq O_2_/kg fat after 15 months of storage, without developing strange odours or flavours. As can be seen, the shelf life of WCh is considerably bigger than the period of analysis possible for this research. However, it can be inferred that the quality of the WChs produced was maintained throughout the 15 days as the TOTOX values did not exceed the maximum established limit of 30 mEq O_2_/kg [12], and the PV did not surpass 10 mEq O_2_/kg, which is considered the starting point for alterations in fat [25].

The present study confirmed that fortifying white chocolate with antioxidants derived from *M. oleifera* leaf extract can help to delay natural lipid oxidation and enhance the oxidative stability of the product. Moreover, MOLE shows potential as a substitute for synthetic antioxidants in WCh without compromising its stability while also improving its biological properties.

## 4. Conclusions

This study aimed to explore the incorporation of *M. oleifera* leaf extract into white chocolate as a possible strategy to produce a fortified product enriched in antioxidants and bioactive compounds. The obtained *M. oleifera* leaf extract (MOLE) exhibited promising antioxidant properties and the ability to inhibit the activity of two enzymes associated with carbohydrate digestion: α-amylase and β-glucosidase. This inhibition is often linked to diabetes management as it slows down carbohydrate metabolism and helps to regulate blood glucose levels. Finally, the primary phenolic compound identified in MOLE was catechin. Four white chocolate samples were formulated, and their microbial safety after production was unaffected by fortification with *M. oleifera*. However, the incorporation of the extract affected the visual aspect of the chocolates, with a higher concentration of MOLE resulting in a more intense green colouration. Future work could test the encapsulation of the extract as a strategy to mask the green colour of the extract in white chocolate. The sample fortified with 3% MOLE exhibited the highest antioxidant capacity after 15 days of storage. Oxidative stability tests indicated that the addition of *M. oleifera* could retard the primary autoxidation of lipids to some extent, with higher concentrations of the extract leading to a slower increase in the oxidation value. Testing higher fortification percentages could be beneficial for enhancing the antioxidant properties and improving the oxidative stability of fortified white chocolates. In addition, microencapsulation might help to prolong these properties over time if a sustained release of the extract can be achieved. It is also important to assess the sensory properties of the fortified chocolates to understand the substitution level limit that does not impair the consumers’ general acceptability of the product. Overall, this research highlights the potential of using phenolic-rich extracts from *M. oleifera* to enhance the biological properties of food, resulting in value-added sustainable products. This approach addresses a critical issue in food science by enabling the production of food with longer shelf life while also tackling nutritional deficiencies and promoting health benefits.

## Figures and Tables

**Table 1:** Ingredients of the different formulations of the white chocolate produced.

Condition	m_cocoa butter_ (g)	m_powder milk_ (g)	m_powder sugar_ (g)	m_MOLE_ (g)	m_lecithin_ (g)
WCh-NC	12	12	6	0	0
WCh-PC	12	11.97	6	0	0.03
WCh-MOLE 1%	12	11.70	6	0.3	0
WCh-MOLE 3%	12	11.10	6	0.9	0

MOLE: *M. oleifera* leaf extract; WCh-NC: white chocolate negative control; WCh-PC: white chocolate positive control; WCh-MOLE 1%: white chocolate incorporated with *M. oleifera* leaf extract at 1% substitution level; WCh-MOLE 3%: white chocolate incorporated with *M. oleifera* leaf extract at 3% substitution level.

**Table 2:** Results from the antioxidant and anti-enzymatic characterisation of the *Moringa oleifera* leaf extract.

Antioxidant Capacity				Enzyme Inhibition	
IC_50_ (mg/L)		TEAC (mg_TE_/g_DW_)		Inhibition %	
DPPH	ABTS	DPPH	ABTS	α-Amylase	β-Glucosidase
1309.6 ± 18.1	162.0 ± 6.0	3.5 ± 0.1	18.5 ± 0.6	79.9 ± 0.3	98.0 ± 1.7

ABTS: 2,2′-azino-bis(3-ethylbenzothiazoline-6-sulphonic-acid); DW: dry weight; DPPH: 2,2-diphenyl-1-picryl-hydrazyl; IC_50_: extract concentration necessary to inhibit 50% of the radical; TE: Trolox equivalent; TEAC: Trolox equivalent antioxidant capacity.

**Table 3:** Concentration of the main phenolic compounds detected in *Moringa oleifera* leaf extract by HPLC-DAD.

Compound	RT (min)	Concentration (mg_compound_/g_extract_)
Caffeic acid	27.8 ± 0.05	0.056 ± 0.002
(+)-Catechin	23.4 ± 0.05	0.211 ± 0.013
Gallic acid	11.1 ± 0.03	NQ
Procyanidin B2	25.7 ± 0.05	0.003 ± 0.002
Quercetin	48.5 ± 0.04	0.031 ± 0.001

RT: Retention time; NQ: not quantifiable.

## Data Availability

The original contributions presented in this study are included in the article/Supplementary Material. Further inquiries can be directed to the corresponding author.

## Supplementary Materials

The following supporting information can be downloaded at https://www.mdpi.com/article/10.3390/foods14030359/s1. Figure S1. Microbiological analysis of the white chocolate samples, in the week of production (t_0_), on Lauryl Sulphate Agar (LSA) medium (top) and Rose Bengal Chloramphenicol Agar (RBC) medium (bottom). Table S1. Total oxidation values of the different white chocolate samples over time.
