# Supplementary material for: Enhancing the Biological Properties of White Chocolate: Moringa oleifera Leaf Extract as a Natural Functional Ingredient
Source: Foods. 2025 Jan 22;14(3):359. doi: 10.3390/foods14030359 (PMC11816540; doi:10.3390/foods14030359)
Supplement: Supplementary file 1 [file foods-14-00359-s001.zip › foods-3370510-supplementary.pdf]

Enhancing the Biological Properties of White Chocolate: *Moringa oleifera* Leaf Extract as a Natural Functional Ingredient

Sandra M. Gomes, Rita Miranda and Lúcia Santos

Supplementary Material

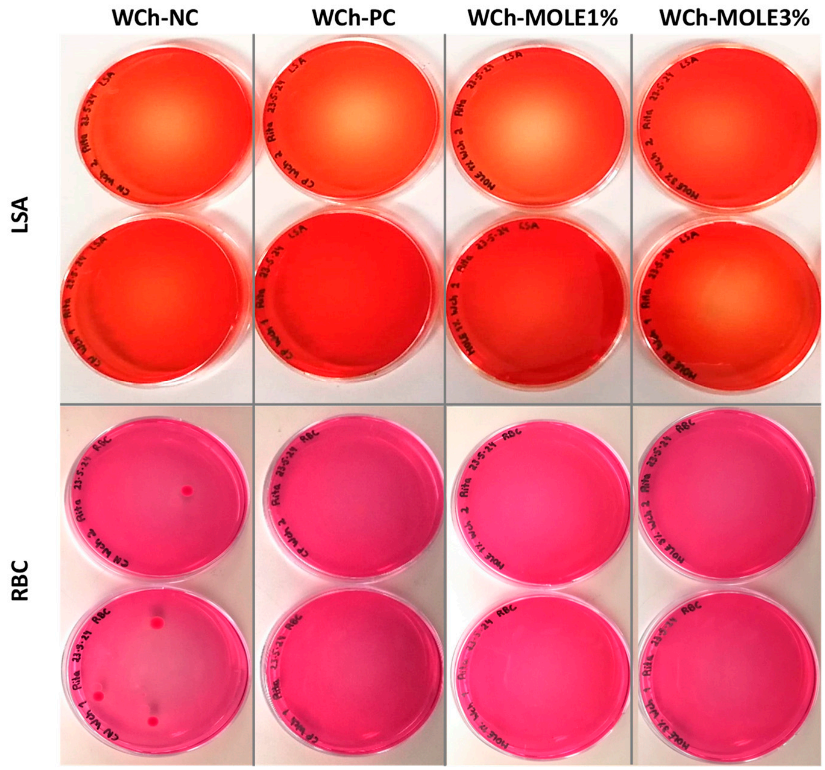

Figure S1. Microbiological analysis of the white chocolate samples, in the week of production (t_0), on Lauryl Sulphate Agar (LSA) medium (top) and Rose Bengal Chloramphenicol Agar (RBC) medium (bottom). From left to right: WCh-NC – white chocolate negative control; WCh-PC – white chocolate positive control; WCh-MOLE1% – white chocolate with *M. oleifera* leaf extract at a substitution level of 1%; and WCh-MOLE3% – white chocolate with *M. oleifera* leaf extract at a substitution level of 3% (Photographs taken on 24/05/2024 and 30/05/2024).

Table S1. Total oxidation values of the different white chocolate samples over time.

	t_0	t_2
**WCh-NC**	1.12	3.73
**WCh-PC**	1.17	3.23
**WCh-MOLE 1%**	1.24	2.75
**WCh-MOLE 3%**	1.07	2.33

The analysis was performed at different timepoints: the week of production, stored in the fridge (t0), and 15 days after storing at room temperature (t2). WCh-NC: white chocolate negative control; WCh-PC: white chocolate positive control; WCh-MOLE 1%: white chocolate incorporated with *M. oleifera* leaf extract at 1% substitution level; WCh-MOLE 3%: white chocolate incorporated with *M. oleifera* leaf extract at 3% substitution level.
